# Agnogenic practices: an analysis of UK gambling industry-funded youth education programmes

**DOI:** 10.1093/eurpub/ckac131.544

**Published:** 2022-10-25

**Authors:** MCI van Schalkwyk, B Hawkins, M Petticrew, A Reeves, M McKee

**Affiliations:** 1 Department of Health Services Research and Policy, LSHTM, London, UK; 2 MRC Epidemiology Unit, University of Cambridge, Cambridge, UK; 3 Department of Public Health, Environments and Society, LSHTM, London, UK; 4 Department of Social Policy and Intervention, University of Oxford, Oxford, UK

## Abstract

**Background:**

The corporate political activities of harmful industries, including the use of agnogenic (ignorance or doubt producing) practices and the construction of dystopian narratives, directed at influencing policymaking are well documented. However, the use of agnogenic practices by industry-funded organisations who deliver industry-favoured education-based measures remains unexplored. This study aims to build understanding of this by analysing three UK gambling industry-funded youth education programmes that represent key policy responses to gambling harms.

**Methods:**

Using a published typology of corporate agnogenic practices the ways that evidence is used within the programmes’ resources to legitimise their content and implementation were analysed. Programme evaluations and claims about the programmes’ evidence base and effectiveness were also analysed.

**Results:**

Agnogenic practices, including confounding referencing, misleading summaries and evidential landscaping, that resemble those adopted by harmful industries are used within gambling industry-funded youth education programmes and by the charities that oversee their delivery. These practices serve corporate interests, distort the limited evidence in support of youth gambling education measures, and legitimise industry favoured policies.

**Conclusions:**

This novel study demonstrates that agnogenic practices are used to construct utopian narratives that claim that gambling industry-favoured youth education programmes are evidence-based and evaluation-led. These practices misrepresent the literature and evaluation findings and may undermine effective policymaking to protect children and young people from gambling harms.

**Key messages:**

• Gambling industry-funded education programmes warrant greater scrutiny and conflicts of interest need to be addressed.

• The methods and findings of this study are of relevance to other contexts and areas in the field of the commercial determinants of health given other harmful industries adopt similar approaches.

